# Clinical Consensus on Diagnosis and Treatment of Patients with Chronic Exertional Compartment Syndrome of the Leg: A Delphi Analysis

**DOI:** 10.1007/s40279-022-01729-5

**Published:** 2022-07-29

**Authors:** Sanne Vogels, E. D. Ritchie, B. L. S. Borger van der Burg, M. R. M. Scheltinga, W. O. Zimmermann, R. Hoencamp

**Affiliations:** 1 Department of Surgery, Alrijne Hospital, Simon Smitweg 1, 2353 GA Leiderdorp, The Netherlands; 2 Trauma Research Unit, Department of Trauma Surgery, Erasmus MC, University Medical Center, Rotterdam, The Netherlands; 3 Department of Surgery, Máxima MC, Veldhoven, The Netherlands; 4 Department of Sports Medicine, Royal Netherlands Army, Utrecht, The Netherlands; 5 Department of Military and Emergency Medicine, Uniformed Services University of the Health Sciences, Bethesda, MD USA; 6 Defense Healthcare Organization, Ministry of Defense, Utrecht, The Netherlands; 7 Department of Surgery, Leiden University Medical Center, Leiden, The Netherlands

## Abstract

**Aim:**

Defining universally accepted guidelines for the diagnosis and treatment of chronic exertional compartment syndrome (CECS) is hampered by the absence of high-quality scientific research. The aim of this Delphi study was to establish consensus on practical issues guiding diagnosis and treatment of CECS of the leg in civilian and military patient populations.

**Methods:**

An international expert group was queried using the Delphi technique with a traditional three-round electronic consultation. Results of previous rounds were anonymously disclosed in the questionnaire of rounds 2 and 3, if relevant. Consensus was defined as > 70% positive or negative agreement for a question or statement.

**Results:**

The panel consisted of 27 civilian and military healthcare providers. Consensus was reached on five essential key characteristics of lower leg CECS. The panel achieved partial agreement regarding standardization of the diagnostic protocol, including muscle tissue pressure measurements. Consensus was reached on conservative and surgical treatment regimens. However, the experts did not attain consensus on their approach of postoperative rehabilitation and preferred treatment approach of recurrent or residual disease. A summary of best clinical practice for the diagnosis and management of CECS was formulated by experts working in civilian and military healthcare facilities.

**Conclusion:**

The Delphi panel reached consensus on key criteria for signs and symptoms of CECS and several aspects for conservative and surgical treatment. The panel did not agree on the role of ICP values in the diagnostic process, postoperative rehabilitation guidelines protocol, or the preferred treatment approach for recurrent or residual disease. These aspects serve as a first attempt to initiate simple guidelines for clinical practice.

## Key Points

**Table:** 

Defining universally accepted guidelines for the diagnosis and treatment of chronic exertional compartment syndrome (CECS) of the leg is hampered by the absence of high-quality scientific research.
A comprehensive overview of current state-of-the-art opinions of a large group of professionals who are deeply involved in the daily care and treatment of CECS patients is provided.
Outcome of this Delphi analysis on CECS may serve as a platform to initiate simple guidelines for clinical practice.

## Introduction

The chronic exertional compartment syndrome (CECS) is considered a disabling overuse injury that may occur in myofascial compartments of lower or upper extremities of active individuals, athletes, or military service members. This syndrome manifests itself upon the performance of repetitive movements and is usually reported as an exercise-related muscular pain, causing a sensation of pressure or tightness that lessens after cessation of provocative activities. These symptoms are thought to result from a reversible elevation in intracompartmental pressures (ICPs), secondary to a mismatch between expansion of muscular tissue within a relatively noncompliant fascia [1]. However, there is increasing evidence that CECS is a multifactorial problem and more than just a temporary elevation of ICP [2]. CECS is mostly encountered in the anterior compartment of the leg. However, reliable incidence or prevalence rates are not available [3, 4].

The diagnostic pathway of CECS starts with a suspicion that is raised after a suggestive patient’s history in combination with painful palpation of affected muscles, ideally immediately after symptom provocation. In clinical practice, an invasive needle or catheter manometry may be used to confirm the presence of CECS [3]. These ICP measurements yield absolute pressure values before, during, and/or after exercise, but diagnostic cut-off criteria differ substantially [2, 5–8]. Moreover, the execution of an ICP measurement suffers from interobserver variability in the absence of a standardized test-protocol [9, 10]. In addition, the invasive nature of these measurements comes with a risk of incorrect needle placement, hematoma formation and nerve damage [11, 12]. Alternative diagnostic tests are currently not widely used in the diagnostic work-up, because the evidence for their use is of low quality or the practicality for clinical use low [13]. Conventional radiographs and MRI scans may be used to exclude alternative diagnoses [3].

The natural course of CECS was shown to cause persistent symptoms over time [14]. Traditionally, management of CECS starts with conservative measures. Gait retraining [15–17] and botulinum injections [18] may have positive outcomes. If conservative interventions fail or if a patient experiences severe symptoms, surgical treatment is considered. Fasciotomy is the described surgical intervention, opening the fascia enveloping the affected muscle with an open, a minimally invasive, or an endoscopic technique [4]. Moreover, the partial removal of fascia (fasciectomy) is used in some military populations [19], and is advised in cases of residual or recurrent disease [20]. However, a clear treatment algorithm and clinical guideline are not available, whereas presentation of treatment outcomes in the scientific literature is far from standardized [21].

Defining universally accepted guidelines for the diagnosis and treatment of CECS of the leg appears to be hampered by the absence of robust empirical evidence. Randomized controlled trials are currently not available and the level of evidence is often limited to level 3 or 4 [21]. The aim of this Delphi study was to establish consensus on practical issues guiding diagnosis and treatment of CECS in civilian and military patient populations.

## Materials and Methods

### Study Design

Opinions of an international expert group were collected by the Delphi technique as initially developed by Dalkey and Helmer in 1963 [22–26]. The consensus process was conducted with a traditional three-round electronic consultation (Linstone 1978, literature search for statement development) [27] between May 2020 and July 2021. Statements were attained regarding diagnosis and management of CECS of the leg in both (recreational) athletes and military service members. An online survey platform was used (SurveyMonkey®, Momentive inc., San Mateo, CA, USA) and individualized links were sent to all participants. Anonymity was warranted for all rounds. Consensus was defined prior to the investigation and fixed at a response rate of > 70% of the panel and a positive or negative agreement of > 70% for a question or a statement [26, 27].

### Panel Selection

Panel participants were considered experts on CECS of the leg on the basis of their scientific track record by the authors. Sports medicine physicians, surgeons, clinical investigators, and physiotherapists actively treating patients with exercise-related leg pain in civilian and/or military patient populations were considered eligible members for an international study group. A portion of the panel members was suggested by all co-authors based on these criteria. Additionally, all invited panelists were asked to suggest potential panel members as well. Upon invitation, a clear explanation of the objectives of the study and specific instructions for member participation were provided and consent was obtained. All panel members were asked to confirm their expertise with respect to the diagnosis and management of CECS, as well as to estimate their annual case load of ICP measurements and/or surgeries.

### Search Strategy, Data Extraction, and Statement Development

The literature search was conducted in PubMed, EMBASE, Web of Science, Cochrane, CENTRAL, and Emcare using the keywords “chronic exertional compartment syndrome,” “anterior compartment,” “posterior compartment,” “peroneal compartment,” “exertional leg pain,” “medial tibial pain,” “overuse injuries,” “diagnosis,” “therapy,” “surgical treatment,” and “conservative treatment.” All related MeSH terms, synonyms and plurals were entered. Studies published between 1 January 1970 and 1 May 2020 were eligible. In addition, relevant publications identified outside this strategy were added manually, based upon recommendations by co-authors.

A core group with the study’s facilitator (SV) and primary researchers was formed. The facilitator screened all titles and abstracts for relevance. Articles were included if they defined, described, or recommended appropriate clinical information related to CECS of the leg, including (1) history-taking questions that aided in the differential diagnosis of exercise-related leg pain; (2) physical examination and special tests; (3) indications for and types of diagnostic investigations; and (4) treatment interventions. All members of the core group independently performed a second screening to verify the completeness of the initial list. Additional relevant publications were provided by the core group. Full texts of studies were retrieved and reviewed for eligibility.

After extensive review of full-text publications, a structured questionnaire for the first Delphi round was developed by the study’s facilitator and a member of the expert panel. Relevant questions were formulated from included studies and covered best practices on how to diagnose and manage patients presenting with possible CECS of the leg. The list of questions was evaluated by the core group independently. Each member grouped questions according to themes to develop the final list of questions. Differences were resolved by discussion. All items and questionnaires were tested by all co-authors to identify ambiguities and to improve on feasibility of administration [23].

### Round 1

The first questionnaire consisted of 28 multiple choice questions. Questions covering a specific statement were answered using an ordinal 5-point Likert scale (strongly agree, agree, neither agree nor disagree, disagree, strongly disagree). Questions with nominal answer categories were provided with the opportunity for comments and suggestions in an open text box. Response frequencies for each item were calculated and entered anonymously into a database by the study’s facilitator. Ordinal or nominal answer categories with > 70% agreement from the panel were accepted and omitted from the development of questions or statements for the subsequent rounds. Statements not meeting a 70% agreement were modified according to feedback provided by the expert panel and redistributed to the panel members for round 2.

### Round 2

A second questionnaire containing 22 multiple choice questions was created with the purpose to further specify the answers of the panel members. Experts were now asked to judge statements with “agree” or “disagree,” or to answer questions with fewer possible nominal answer categories. Throughout this round, all panel members were informed on the summarized scores and comments that were obtained in round 1. Thus, panel members could reflect upon the group results and adjust their opinion, while preserving the anonymity of their responses. Questions and statements not reaching consensus were retained for discussion in round 3.

### Round 3

This final questionnaire consisted of 11 questions. As only a small increase in degree of consensus was expected during this stage of the survey, four open questions were implemented to clarify dispersion in previous answers. The panel members were asked to revise previous answers or to specify reasons for remaining outside of the consensus. Non-responders in rounds 1 and 2 were excluded from this final round.

### Statistical Analysis

Statistical analysis was executed using SPSS statistics (v26, IBM Corporation, Armonk, NY, USA). All the panel members answers were registered in an electronic data file provided by the online survey platform. Descriptive statistics were used to present the data by frequencies (percentage). A sub-analysis comparing non-surgical with surgical panel members was performed for the questions regarding recurrent or residual disease, using Fisher’s exact test. For this analysis, *p* values (two-sided) ≤ 0.05 were considered significant.

## Results

### Delphi Panel Members

A total of 40 experts were identified and invited by email to join as a panel member. Seven potential panel members did not participate due to technical reasons (18%; digital invitations bounced *n* = 3, remained unopened *n* = 4). Another six candidates did not consider themselves an expert. Therefore, the international Delphi consensus panel was performed with 27 civilian and military care providers (sports medicine physician *n* = 12, surgeon *n* = 12, physiotherapist *n* = 2, and clinical investigator *n* = 1; Table 1). The first round was completed by all panel members, whereas rounds 2 and 3 were completed by 24 members (89%). The majority of the panel members were involved with civilian patient populations (89%) and were located in Europe (67%; Table 1).

**Table 1:** Demographics of the Delphi panel members (*n* = 27)

	*n* (%)
Gender	
Male	26 (96)
Female	1 (4)
Profession	
Sports medicine physician	12 (44)
Orthopedic surgeon	6 (22)
Vascular surgeon	3 (11)
Trauma surgeon	3 (11)
Physiotherapist	2 (7)
Clinical researcher	1 (4)
Place of practice	
Europe	18 (67)
North America	9 (33)
Patient population	
Civilian	19 (70)
Military	3 (11)
Both	5 (19)
Intracompartmental pressure measurements in current practice, per year	
< 10	8 (30)
10–29	5 (19)
30–49	5 (19)
50–99	4 (15)
> 100	5 (19)
Surgical procedures in current practice, per year	
< 10	11 (41)
10–29	7 (26)
30–49	3 (11)
50–99	1 (4)
> 100	1 (4)
Unknown	4 (15)

### Diagnosis of Chronic Exertional Compartment Syndrome

#### Patient’s History

The panel members agreed that both pain (25/27; 93%) and tightness (24/27; 89%) during exercise are essential clues in the history of patients suspected of CECS. Additionally, the specific location of symptoms (27/27; 100%), activity modification (22/27; 81%), and the type of provocative activity (20/27; 74%) were considered conditional aspects of a patient’s history. The provocative activity was further specified in the second round as “involvement in sports or activities that require repetitive activation of the same muscles,” to which all panel members agreed as conditional (24/24; 100%).

Cramping, weakness, and paresthesia during exercise were not considered essential symptoms (21/24; 88%). In addition, duration of symptoms in the leg was not considered conditional for the diagnosis (21/14; 88%).

#### Physical Examination

Initially, no signs upon physical examination were considered essential for diagnosis, although “pain induced by treadmill provocation” was close to consensus (18/27; 67%). Following suggestions from an open textbox, this statement was changed to “symptoms induced by provocative activities” for the second round. This change in wording was considered essential by 82% (22/24) of the panel. Signs such as tenderness upon palpation of the symptomatic compartment (10/27; 37%) or presence of a muscle herniation (6/27; 22%) were not considered essential.

The panel did not reach consensus on any system used for the scoring of symptoms during physical examination or symptom provocation. Approximately half of the panel preferred a Visual Analogue Scale (14/24; 52%) or a Numeric Rating Scale (13/24; 48%). Moreover, the proposal to use either a Visual Analogue Scale or a Numeric Rating Scale in the absence of a standardized scoring system for CECS symptoms did not reach consensus either (18/24; 67%).

#### Intracompartmental Pressure Measurements

The panel agreed that an ICP measurement is conditional for the diagnosis (22/27; 82%; Textbox 1). In addition, five panel members (21%) indicated that ICPs should be used complementary to clinical signs and symptoms, due to the existing variation in measurement protocols and cut-off values. Another part of the panel indicated that ICP measurements are used to differentiate between pathological etiologies responsible for exercise-related leg pain (3/24; 13%) or to establish the indication for surgical treatment (3/24; 13%).

**Table:**
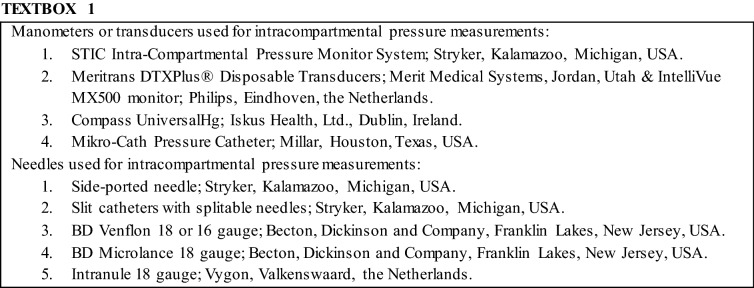

It was agreed upon that the ICP value obtained 1 min after a provocative exercise is most meaningful and therefore preferred (20/24; 80%). The panel also thought that ICP measurements ought to be performed in symptomatic compartments of both legs (17/24; 71%). Where the deep posterior compartment is measured, a medial approach is preferred (21/24; 87%), whereas correct tip placement using ultrasound is required (17/24; 71%).

The panel did not achieve consensus with respect to leg and patient positioning. In the first round, supine positioning was most popular (19/27; 71%), although panel members also preferred a standing (5/27; 19%) or a sitting position (7/27; 26%). In the second round this question was rephrased as “are ICP measurements best performed supine compared to sitting or standing.” This statement was agreed upon by 16 of the 24 panel members (67%). Reasons to perform an ICP measurement in a standing position included: (1) a supine position would falsely decrease the ICP by counteracting the effect of gravity, (2) a standing position is similar to the provocative situation, or (3) local diagnostic criteria were developed using a standing position.

Another aspect without consensus was the ICP cut-off value. The majority of panel members either used the Pedowitz criteria [8] or part of these (13/24; 54%). Nine panel members (38%) indicated they were using locally established cut-off values, or ICP values different from the Pedowitz criteria [2, 5, 7].

#### Alternative Diagnostic Tests

Aside from ICP measurements, the panel considered “symptom provocation by use of a treadmill test and repeat physical examination” a conditional test for the diagnosis (21/27; 78%). The majority of the panel did not use additional diagnostic examinations (e.g., conventional radiography, computerized tomography scans, or magnetic resonance imaging) to confirm the presence of CECS (23/24; 96%).

#### Diagnosis of Chronic Exertional Compartment Syndrome

Panel members reached consensus that signs and symptoms are the essential aspects of the diagnostic work-up (23/26; 88%). Based on the questions covering the diagnostic work-up in rounds 1 and 2 of the Delphi procedure, five key criteria were formulated (Textbox 2). All panel members considered these criteria key to the clinical diagnosis of CECS (24/24; 100%). In addition, six members (25%) suggested adding the reversible or transient character of CECS symptoms to this list. More specifically, they claimed that symptoms resolved within minutes of rest, rather than hours or days. After introduction of the key criteria in round 3, ICP measurements were still regarded essential by 38% of the panel (9/24). Three of these panel members indicated that if more evidence was available regarding the diagnostic accuracy (i.e., sensitivity and specificity) of these criteria, the measurement of ICPs might become non-essential.

**Table:**
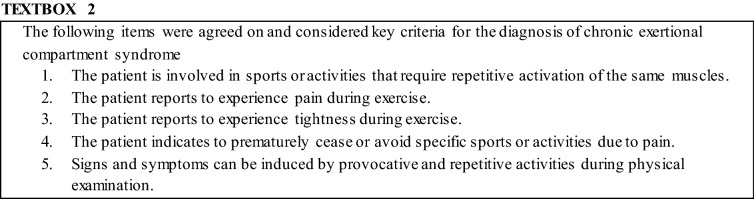

### Management of Chronic Exertional Compartment Syndrome

#### Conservative Treatment

The panel agreed that gait retraining (23/26; 88%) and cessation of provoking activities (17/24; 71%) are valuable components of a conservative treatment program. The panel considered guidance and treatment by a physical therapist (15/24; 63%), shoe modifications (13/24; 54%), or botulinum injections (5/24; 21%) not essential for a conservative treatment regimen, but rather as potential additions depending on patient needs.

#### Surgical Treatment

**Fig. 1:**
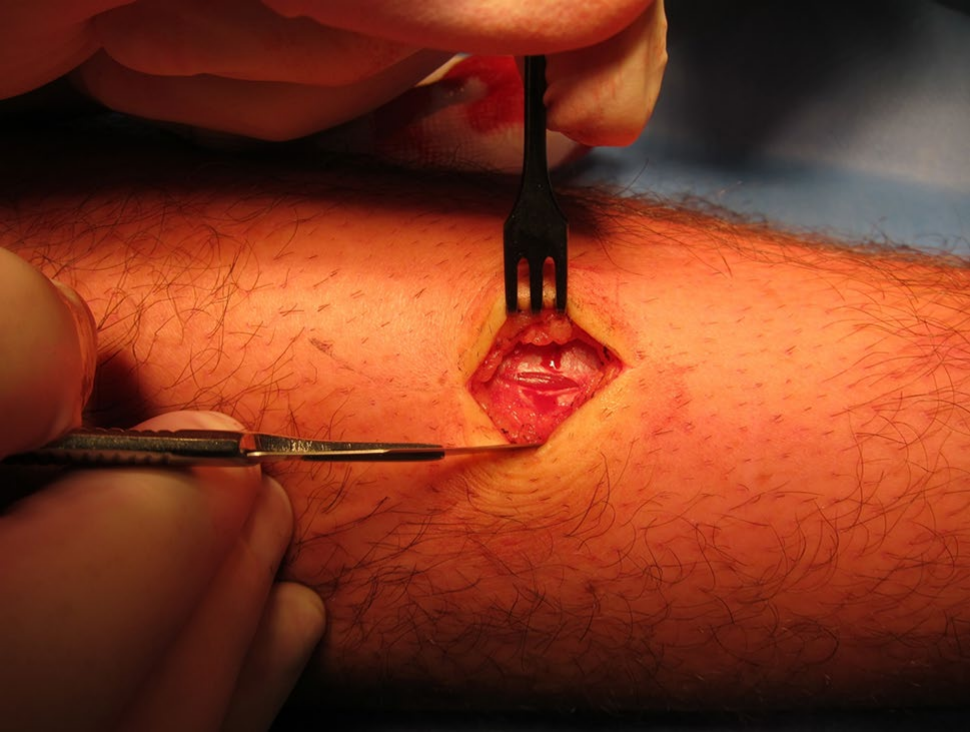
Consensus was attained regarding a minimally invasive fasciotomy for CECS of the lower leg anterior compartment including a single 2-cm longitudinal skin incision for introduction of the fasciotome (see also Textbox 3)

The surgeons in the panel agreed that a fasciotomy of the anterior compartment can be performed safely and effectively using the entrance of a single small incision, 1–2 cm laterally from the tibial crest, at the distal two-thirds of the lower leg (10/12; 83%; Fig. 1). The surgical panel did not agree on the type of fasciotome that was required for the minimally invasive fasciotomy (Textbox 3). Additionally, they also agreed that a fasciotomy of the lateral compartment can be performed safely and effectively using two incisions, each 5–8 cm in length, located in between the fibular head and the lateral malleolus (11/12; 92%). In case both compartments require opening during the same session, 83% (10/12) agreed this can be done safely and effectively using a single 4- to 6-cm incision, at the intramuscular septum between the anterior and lateral compartment. No consensus was reached on whether or not compartments other than the affected ones require preventive surgery [for instance, the lateral compartment in case only the anterior compartment was affected (4/12; 33%) or vice versa (5/12; 42%)].

**Table:**
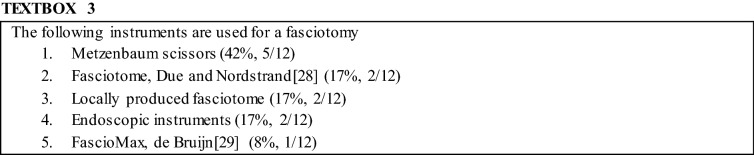

The panel agreed on the statement that the deep posterior compartment should be approached medially using a large incision (11/12; 92%) and that the superficial posterior compartment should be opened prior to approaching the deep posterior compartment (8/12; 80%). Only half of the surgeons would actively look for and subsequently open deep posterior sub-compartments (6/12; 50%).

Visualization and localization of the superficial peroneal nerve during fasciotomy of the anterior compartment remains a topic of discussion, as only 42% (5/12; round 1) and 67% (8/12; round 2) of the surgeons considered this necessary. Moreover, ultrasound was not considered an effective tool for visualization of the superficial peroneal nerve (5/12; 42%).

No consensus was reached on the instruments that should be used during surgery. An overview of suggested instruments can be found in Textbox 3.

#### Postoperative Rehabilitation

Consensus was reached on the statement that a standardized institutional rehabilitation protocol should be used postoperatively (21/24; 88%), not necessarily adjusted for the compartment requiring the fasciotomy (17/24; 71%). However, the panel members could not agree on the type of restrictions in such a protocol. Permissive weight bearing was allowed according to 67% (16/24), whereas 33% (8/24) favored unrestricted weight bearing. In the first 2 weeks, sports participation is not allowed by 46% (11/24), but permitted if not painful by 50% (12/24).

### Treatment Outcome

The panel agreed that standardization of outcome measures is preferred (100%, 24/24). They agreed that the treatment outcome should be monitored using the parameters “Return to previous level of sports” [round 1: 23/26 (89%); round 2: 22/24 (92%)], “Lower, similar, or higher level of physical activity” [round 1: 19/26 (73%); round 2: 23/24 (96%)], and the “Global Perceived Effect scale” [28] [round 1: 11/26 (42%); round 2: 19/24 (83%)].

### Recurrent Disease

No consensus was achieved on management of recurrent or residual disease after surgical treatment. In case of recurrent CECS, panel members considered the following actions: conservative treatment [anterior compartment 6/23 (26%); lateral compartment 7/24 (29%); deep posterior compartment 9/24 (38%)], a re-fasciotomy [anterior compartment 3/23 (13%); lateral compartment 6/24 (25%); deep posterior compartment 7/24 (29%)], a four-compartment fasciotomy [anterior compartment 2/23 (9%); lateral compartment 2/24 (8%); deep posterior compartment 2/24 (8%)], and a fasciectomy [anterior compartment 12/23 (52%); lateral compartment 9/24 (38%); deep posterior compartment 6/24 (25%)]. This variation in answers was comparable amongst non-surgical and surgical panel members (anterior compartment *p* = 0.11; lateral compartment *p* = 0.11, posterior compartment *p* = 0.62).

## Discussion

This three-round Delphi analysis established consensus on several aspects regarding the clinical practice guidelines for diagnosis and treatment of lower leg CECS in both civilian and military patient populations. A panel with 27 international expert members reached consensus on five key sign and symptom criteria of CECS. Cessation of provocative activities and gait retraining were considered valuable components for a conservative treatment program. All surgeons on the panel agreed on the size and location of the incision for the fasciotomy. No consensus was reached on the diagnostic role of muscle compartment pressures (ICP). Additionally, opinions on postoperative rehabilitation protocol and treatment of recurrent or residual CECS varied considerably, although there was consensus these aspects should be standardized.

Current diagnosis and management of patients with CECS occurs in the absence of universally accepted guidelines and standardized protocols. Earlier literature already identified a large heterogeneity in evidence on diagnosis [5, 6] and treatment [21, 29] of CECS. The current study is a first attempt forwarded by expert-based evidence initiating simple guidelines for clinical practice. The experienced panel members are considered leading figures in the field of exercise-related leg pain having declared ample work experience with CECS. The international character of the panel lends credit to the statements on which consensus was achieved.

The most prominent result of the current study was the consensus attained on the five proposed key criteria summarizing crucial signs and symptoms of CECS (Textbox 2). Several studies attempted to identify prognostic clinical factors reflecting diagnosis of CECS. Examples are post-effort muscle hardness and presence of a fascial hernia [30, 31]. Also, participation in running or skating, pain recidivism upon performing the same exercise, and the absence of pain at rest were also mentioned [31]. The panel members of the current study agreed that CECS was considered likely if the patient (1) is involved in activities that require repetitive activation of the same muscle(s), (2) reports pain during exercise, (3) reports tightness during exercise, (4) prematurely ceases or avoids specific activities, and (5) can induce symptoms by performing provocative activities during physical examination. Contrary to the previously published literature is the lack of consensus amongst the panel members with regard to tenderness upon palpation over the affected compartment or the presence of a muscle herniation (37% and 22%, respectively). The expert panel also indicated that, aside from the ability to induce symptoms by provocative activities, no other signs were considered essential for the diagnosis of CECS. Future evaluation of the currently proposed key criteria, in combination with previous literature, can aid in the development of a proficient screening tool for daily clinical practice.

Panel members did not reach consensus on the role and execution of ICP measurements in the diagnostic process of CECS. This disagreement could reflect the ongoing debate in current literature, in which the value of test protocols and cut-off values are repeatedly questioned [5, 6, 9, 32]. The panel achieved consensus on the preferred timing of ICP measurement (1 min after completion of a symptom provocation test), number of legs and compartments (bilateral, only affected compartments), and route of catheter administration in suspected deep posterior compartment (medial approach). Yet, the ongoing lack of consensus on cut-off values and patient positioning is worrisome. However, several members of the panel reported that ICP values could be considered as complementary or non-essential once the anamnestic key criteria strongly suggest CECS. If the proposed set of specific key criteria is validated or alternative non-invasive tests become available, invasive ICP measurements will become obsolete.

The postoperative rehabilitation protocol and treatment of recurrent (or residual) disease necessitates future research. The lack of agreement amongst rehabilitation protocols was already identified in previous literature [3]. For example, surgeons encourage early mobilization of the limb in an attempt to reduce scar tissue formation [33]. However, the timing of mobilization or initiation of exercises varies in current literature from directly after surgery to somewhere in the first 7 days [34–39]. Also, the use of analgesics is not univocally prescribed in these studies. This lack of agreement was confirmed by current findings, as no consensus could be reached on weight bearing and sports participation after surgical treatment. The panel, however, was not specifically tabulated on the encouragement of early mobilization or the use of analgesics throughout rehabilitation. In addition, the experts did not agree on the most appropriate approach for recurrent or residual disease after surgical treatment. Interestingly, this variation in answers was present amongst both non-surgical and surgical physicians. The variation in guidelines in current literature, in combination with the absence of consensus in the current study indicate that future research should also focus on these specific issues of postoperative rehabilitation.

The current study has limitations that characterize every Delphi analysis. First, selection of the Delphi panel members was dependent on the scientific network and subjective judgment of the authors. Bias might have been introduced by the exclusion of potential panel members due to technical reasons (18%). Secondly, although this analysis method is an accepted methodology for gaining level 5 evidence, the present findings require confirmation in future research. The technique is based on expert opinions and does not replace scientific reports with original research. Also, the currently used 70% threshold level of consensus is debated as a scientific rationale is not available [27]. Nevertheless, the present study does provide a comprehensive overview of current state-of-the-art opinions of a large group of professionals who are deeply involved in the daily care and treatment of CECS patients.

## Conclusion

An international multidisciplinary expert panel reached consensus on five key characteristics for the diagnosis of CECS in the leg. The panel achieved partial agreement on statements regarding ICP measurements and conservative and surgical treatment of CECS. No consensus was reached with respect to postoperative rehabilitation guidelines, nor the preferred treatment for recurrent or residual disease. The outcome of this Delphi analysis on CECS may serve as a platform to initiate simple guidelines for clinical practice.

## Abbreviations

CECS: Chronic exertional compartment syndrome
ICP: Intracompartmental Pressure

## Declarations

### Availability of data and material

Anonymous participant data is available via the corresponding author (SV) upon reasonable request.

### Disclaimer

The opinions or assertions contained herein are the private views of the authors and are not to be construed as official or reflecting the views of the Department of Defense of the Netherlands or USA. Several authors and members of the expert panel are current or previous government employees.

## References

[1] Wilder RP, Magrum E (2010). Exertional compartment syndrome. Clin Sports Med.

[2] Zimmermann WO, Ligthert E, Helmhout PH, Beutler A, Hoencamp R, Backx FJG (2018). Intracompartmental pressure measurements in 501 service members with exercise-related leg pain. Transl J Am Coll Sports Med.

[3] Vajapey S, Miller TL (2017). Evaluation, diagnosis, and treatment of chronic exertional compartment syndrome: a review of current literature. Phys Sportsmed.

[4] Buerba RA, Fretes NF, Devana SK, Beck JJ (2019). Chronic exertional compartment syndrome: current management strategies. Open Access J Sports Med.

[5] Aweid O, Del BA, Malliaras P, Iqbal H, Morrissey D, Maffulli N (2012). Systematic review and recommendations for intracompartmental pressure monitoring in diagnosing chronic exertional compartment syndrome of the leg. Clin J Sport Med.

[6] Roberts A, Franklyn-Miller A (2012). The validity of the diagnostic criteria used in chronic exertional compartment syndrome: a systematic review. Scand J Med Sci Sports.

[7] Roscoe D, Roberts AJ, Hulse D (2015). Intramuscular compartment pressure measurement in chronic exertional compartment syndrome: new and improved diagnostic criteria. Am J Sports Med.

[8] Pedowitz RA, Hargens AR, Mubarak SJ, Gershuni DH (1990). Modified criteria for the objective diagnosis of chronic compartment syndrome of the leg. Am J Sports Med.

[9] Hislop M, Tierney P (2011). Intracompartmental pressure testing: results of an international survey of current clinical practice, highlighting the need for standardised protocols. Br J Sports Med.

[10] Large TM, Agel J, Holtzman DJ, Benirschke SK, Krieg JC (2015). Interobserver variability in the measurement of lower leg compartment pressures. J Orthop Trauma.

[11] Winkes MB, Tseng CM, Pasmans HL, Cruijsen-Raaijmakers M, Hoogeveen AR, Scheltinga MR (2016). Accuracy of palpation-guided catheter placement for muscle pressure measurements in suspected deep posterior chronic exertional compartment syndrome of the lower leg: a magnetic resonance imaging study. Am J Sports Med.

[12] Haig AJ, Goodmurphy CW, Harris AR, Ruiz AP, Etemad J (2003). The accuracy of needle placement in lower-limb muscles: a blinded study. Arch Phys Med Rehabil.

[13] Ritchie ED, Vogels S, Van Dongen TTCF, Borger van der Burg BLS, Scheltinga MRM, Zimmermann WO, et al. Systematic review of innovative diagnostic tests for chronic exertional compartment syndrome in the lower leg. Submitted. 2022. 10.1055/a-1866-5957 PMC9815949 35649437

[14] Van der Wal WA, Heesterbeek PJ, Van den Brand JG, Verleisdonk EJ (2015). The natural course of chronic exertional compartment syndrome of the lower leg. Knee Surg Sports Traumatol Arthrosc.

[15] Diebal AR, Gregory R, Alitz C, Gerber JP (2012). Forefoot running improves pain and disability associated with chronic exertional compartment syndrome. Am J Sports Med.

[16] Helmhout PH, Diebal AR, van der Kaaden L, Harts CC, Beutler A, Zimmermann WO (2015). The effectiveness of a 6-week intervention program aimed at modifying running style in patients with chronic exertional compartment syndrome: results from a series of case studies. Orthop J Sports Med.

[17] Zimmermann WO, Hutchinson MR, Van den Berg R, Hoencamp R, Backx FJG, Bakker EWP (2019). Conservative treatment of anterior chronic exertional compartment syndrome in the military, with a mid-term follow-up. BMJ Open Sport Exerc Med.

[18] Isner-Horobeti ME, Muff G, Lonsdorfer-Wolf E, Deffinis C, Masat J, Favret F (2016). Use of botulinum toxin type A in symptomatic accessory soleus muscle: first five cases. Scand J Med Sci Sports.

[19] Roberts AJ, Krishnasamy P, Quayle JM, Houghton JM (2015). Outcomes of surgery for chronic exertional compartment syndrome in a military population. J R Army Med Corps.

[20] Vogels S, van Ark W, Janssen L, Scheltinga MRM. Fasciectomy for recurrent chronic exertional compartment syndrome of the anterior leg. Med Sci Sports Exerc. 2021. 10.1249/MSS.0000000000002631 33731658

[21] Vogels S, Ritchie ED, van Dongen TTCF, Scheltinga MRM, Zimmermann WO, Hoencamp R (2020). Systematic review of outcome parameters following treatment of chronic exertional compartment syndrome in the lower leg. Scand J Med Sci Sports.

[22] Dalkey N, Helmer O (1963). An experimental application of the delphi method to the use of experts. Manage Sci.

[23] Powell C (2003). The Delphi technique: myths and realities. J Adv Nurs.

[24] Meshkat B, Cowman S, Gethin G, Ryan K, Wiley M, Brick A (2014). Using an e-Delphi technique in achieving consensus across disciplines for developing best practice in day surgery in Ireland. J Hosp Admin.

[25] Trevelyan EG, Robinson PN (2015). Delphi methodology in health research: how to do it?. Eur J Integr Med..

[26] Hsu C-C, Sandford B. The Delphi technique: making sense of consensus. Practical assessment, research and evaluation. 2007;12.

[27] Keeney S, Hasson F, McKenna H (2006). Consulting the oracle: ten lessons from using the Delphi technique in nursing research. J Adv Nurs.

[28] Kamper SJ, Ostelo RWJG, Knol DL, Maher CG, de Vet HCW, Hancock MJ (2010). Global Perceived Effect scales provided reliable assessments of health transition in people with musculoskeletal disorders, but ratings are strongly influenced by current status. J Clin Epidemiol.

[29] Rajasekaran S, Hall MM (2016). Nonoperative management of chronic exertional compartment syndrome: a systematic review. Curr Sports Med Rep.

[30] de Bruijn JA, van Zantvoort APM, van Klaveren D, Winkes MB, Cruijsen-Raaijmakers M, Hoogeveen AR (2018). Factors predicting lower leg chronic exertional compartment syndrome in a large population. Int J Sports Med.

[31] Fouasson-Chailloux A, Menu P, Allorent J, Dauty M (2018). Determination of the predictive clinical parameters to diagnose chronic exertional compartment syndrome. Eur J Sport Sci.

[32] Cruz AI, Laidlaw MS (2015). Invasive compartment pressure testing for chronic exertional compartment syndrome: a survey of clinical practice among military orthopedic surgeons. Am J Orthop (Belle Mead NJ).

[33] Tzortziou V, Maffulli N, Padhiar N (2006). Diagnosis and management of chronic exertional compartment syndrome (CECS) in the United Kingdom. Clin J Sport Med.

[34] Biedert RM, Marti B (1997). Intracompartmental pressure before and after fasciotomy in runners with chronic deep posterior compartment syndrome. Int J Sports Med.

[35] Mouhsine E, Garofalo R, Moretti B, Gremion G, Akiki A (2006). Two minimal incision fasciotomy for chronic exertional compartment syndrome of the lower leg. Knee Surg Sports Traumatol Arthrosc.

[36] Schepsis AA, Martini D, Corbett M (1993). Surgical management of exertional compartment syndrome of the lower leg. Long-term followup. Am J Sports Med.

[37] Slimmon D, Bennell K, Brukner P, Crossley K, Bell SN (2002). Long-term outcome of fasciotomy with partial fasciectomy for chronic exertional compartment syndrome of the lower leg. Am J Sports Med.

[38] Irion V, Magnussen RA, Miller TL, Kaeding CC (2014). Return to activity following fasciotomy for chronic exertional compartment syndrome. Eur J Orthop Surg Traumatol.

[39] Turnipseed WD (2002). Diagnosis and management of chronic compartment syndrome. Surgery.

